# Glutamine Promotes Myogenesis in Myoblasts Through Glutaminolysis-Mediated Histone H3 Acetylation That Enhances Myogenin Transcription

**DOI:** 10.3390/nu17233673

**Published:** 2025-11-24

**Authors:** Masaru Takatoya, Tomoya Kasugai, Daichi Arai, Urara Kasuga, Chisato Miyaura, Michiko Hirata, Yoshifumi Itoh, Tsukasa Tominari, Yoshitsugu Aoki, Masaki Inada

**Affiliations:** 1 Cooperative Major of Advanced Health Science, Tokyo University of Agriculture and Technology, 2-24-16 Naka-cho, Koganei 184-8588, Tokyo, Japan; 2 Department of Biotechnology and Life Science, Tokyo University of Agriculture and Technology, 2-24-16 Naka-cho, Koganei 184-8588, Tokyo, Japanmiyaura@isc.chubu.ac.jp (C.M.); hirata@cc.tuat.ac.jp (M.H.); tominari@ncnp.go.jp (T.T.); 3 Inada Research Unit, Institute of Global Innovation Research, Tokyo University of Agriculture and Technology, 2-24-16 Naka-cho, Koganei 184-8588, Tokyo, Japan; yoshi.itoh@kennedy.ox.ac.uk; 4 Kennedy Institute of Rheumatology, Nuffield Department of Orthopaedics, Rheumatology and Musculoskeletal Sciences, University of Oxford, Oxford OX3 7FY, UK; 5 Department of Molecular Therapy, National Institute of Neuroscience, National Center of Neurology and Psychiatry, Kodaira 187-8502, Tokyo, Japan; tsugu56@ncnp.go.jp

**Keywords:** glutamine, C2C12 myoblast, epigenetic regulation, glutaminolysis, histone H3 acetylation

## Abstract

**Background/Objectives:** Plasma glutamine levels in skeletal muscle change in response to exercise intensity and duration, both in physiological and pathological states. Glutamine contributes to muscle differentiation and regeneration; however, the mechanisms underlying this process remain unclear. This study investigated the role of glutamine glutaminolysis in myogenic differentiation, with a focus on epigenetic regulation of myogenin gene expression. **Methods:** C2C12 myoblasts were differentiated into myotubes using media containing various concentrations of glutamine, glutamate, or dimethyl 2-oxoglutarate (DM-α-KG), a cell-permeable analog of α-ketoglutarate. **Results:** Glutamine, glutamate, and DM-α-KG promoted C2C12 myoblast differentiation in a concentration-dependent manner, whereas the glutaminase inhibitor CB-839 suppressed differentiation. 4 mM glutamine increased myogenin mRNA expression by about 5-fold. CB-839 also inhibited glutamine-induced expression of myogenin but did not influence the effects of glutamate or DM-α-KG. Furthermore, glutamine increased histone H3 lysine 27 acetylation (H3K27ac) by about two-fold, whereas CB-839 (200 nM) and A-485 (10 µM), a CBP/p300 histone acetyltransferase inhibitor, reduced H3K27ac levels by about half. These results indicate that glutamine not only serves as a structural amino acid for muscle formation but also enhances myogenin transcription through epigenetic mechanisms. **Conclusions**: This report demonstrates glutaminolysis-dependent histone H3 acetylation, which induces myogenin transcription in myoblasts. These results, connecting glutamine supplementation during resistance training, may make it an effective strategy to accelerate muscle regeneration.

## 1. Introduction

Glutamine (Gln) is the most abundant free amino acid in the human body, with the highest accumulation seen in skeletal muscle [1]. It serves multiple physiological functions, including nitrogen transport and maintenance of acid-base homeostasis, and provides an energy source for immune and intestinal epithelial cells. In skeletal muscle, Gln plays a crucial role in regulating the balance between protein synthesis and degradation [2,3]. Recent studies have shown that plasma Gln levels remain persistently below baseline in athletes with overtraining syndrome, while Gln supplementation alleviates exercise-induced muscle damage in professional athletes, such as basketball players [4,5].

Glutaminase 1 (GLS1) converts Gln to glutamate (Glu), which is further metabolized to α-ketoglutarate (α-KG) by Glu dehydrogenase (GLUD1) in the mitochondria. This metabolic pathway, known as glutaminolysis, produces α-KG, which enters the tricarboxylic acid (TCA) cycle, generating key intermediates such as acetyl-CoA [6]. Acetylation of non-histone proteins and histone proteins within cells reportedly increases in response to elevated intracellular acetyl-CoA concentrations [7,8]. Histone acetyl-CoA transferase p300 has also been reported to follow the Michaelis-Menten equation over an acetyl-CoA concentration range of 3.6–12.5 µM [9]. Based on these studies, increased intracellular acetyl-CoA production leads to elevated p300 enzyme activity, thereby enhancing histone acetylation. Recent evidence indicates that Gln-derived acetyl-CoA contributes to histone acetylation and plays an important role in epigenetic regulation of gene expression [10,11,12].

Muscle regulatory factors (MRFs), including myogenic factor 5 (Myf5), myogenic differentiation 1 (MyoD), myogenin (Myog), and myogenic factor 6 (Mrf4), belong to the basic helix–loop–helix (bHLH) family of transcription factors that govern the commitment and differentiation of skeletal muscle stem cells during myogenesis [13]. Myf5, MyoD, and MRF4 initiate the differentiation of mesodermal progenitor cells into myoblasts, whereas Myog regulates myogenesis in myoblasts, fusion into myotubes, and maturation into multinucleated myofibers [13,14,15,16].

Several in vivo studies have demonstrated that Gln supplementation significantly increases MyoD and Myog expression in skeletal muscles [17,18]. For instance, in a mouse model of cecal ligation and puncture-induced sepsis, oral Gln administration maintained plasma Gln levels and resulted in elevated Myog mRNA expression in skeletal muscle [18]; however, the molecular mechanisms by which Gln regulates the induction of MRFs in vitro remain largely unknown. The molecular mechanisms underlying Gln action in myogenic differentiation are also unclear, although Gln is known to regulate muscle protein synthesis via the mechanistic target of rapamycin (mTOR) signaling pathway and promote muscle regeneration following injury [19,20,21,22,23]. As a current limitation of knowledge, the mechanistic relationship between glutaminolysis and the epigenetic regulation of myogenesis remains largely unclear. Gln contributes to the generation of acetyl-CoA through the TCA cycle, and acetyl-CoA has been reported to promote p300-dependent H3 acetylation at gene promoters.

The present study investigated the effects of Gln on myogenic differentiation and explored the roles of Gln-derived metabolites, such as acetyl-CoA, in glutaminolysis and transcriptional activation of Myog through p300-mediated epigenetic regulation.

## 2. Materials and Methods

### 2.1. Chemicals

Dulbecco’s Modified Eagle Medium with GlutaMAX™ (Cat No. 10569), without Gln (Cat No. 10313), and 100× penicillin streptomycin were purchased from Thermo Fisher Scientific Inc. (Waltham, MA, USA). Fetal bovine serum (FBS) was purchased from Nichirei Bioscience, Inc. (Tokyo, Japan). Horse serum (HS) was purchased from the American Type Culture Collection (Manassas, VA, USA). Cell matrix collagen type I-A was purchased from Nitta Gelatin Inc. (Osaka, Japan). L-Glu, human basic fibroblast growth factor (hbFGF), and dimethyl 2-oxoglutarate (DM-α-KG) were purchased from Sigma-Aldrich, Inc. (St. Louis, MO, USA). CB-839 and A-485 were purchased from Selleck, Inc. (Houston, TX, USA).

### 2.2. Cell Culture

The mouse myoblastic cell line C2C12 was kindly provided by the National Center of Neurology and Psychiatry (Tokyo, Japan). C2C12 cells were maintained as undifferentiated myoblasts in growth media (GM; DMEM [L-Gln: 4 mM], 10% [*v*/*v*] FBS, 1% [*v*/*v*] PS [penicillin streptomycin], 0.1% [*v*/*v*] [2.5 ng/mL] hbFGF) in 100 mm dishes. Prior to plating C2C12 myoblasts, collagen type I-A solution (100 µg/mL in pH 3.0 HCl) was coated in a 24-well plate or 35 mm dish. C2C12 myoblasts were plated in a 24-well plate (1.0 × 10^5^ cells/well) or 35 mm dish (2.5 × 10^5^ cells/dish) coated with collagen type I-A. After incubation of cells in GM for 24 h, myotube formation of C2C12 myoblasts was induced by replacing the medium with differentiation medium (DM; DMEM [L-Gln: 4 mM], 5% [*v*/*v*] HS, 1% [*v*/*v*] PS) and then cultured to form myotubes in DM. The culture medium was changed every other day. To verify the concentration dependence of Gln, DM containing 0, 1, 2, or 4 mM Gln was used for cell cultures. In the inhibitor experiments, either the GLS inhibitor CB-839 or the CBP/p300 inhibitor A-485 was added to DM containing 4 mM Gln. In the Gln metabolite experiments, cells were cultured for 6 h in DM, and then the medium was replaced with DM without Gln but supplemented with Glu or DM-α-KG and/or the respective inhibitors.

### 2.3. Analysis of Myotube Diameter

Analysis of myotube diameter was used to assess the variability of myotube formation. C2C12 myoblasts were cultured in 24-well plates to form the myotubes. Myotubes in each well were microscopically imaged using an inverted microscope (CKX31; OLYMPUS, Tokyo, Japan) with a 4× objective lens and compact digital camera (SP-610UZ; OLYMPUS). Each well bottom was divided into three equal areas, and one microscopic image was captured from each area. Three images were obtained per well, resulting in nine microscopic images per group. The short diameters of all myotubes were measured using ImageJ software (version 1.53e; National Institutes of Health, Bethesda, MD, USA). The short diameter of the myotubes was measured perpendicular to their long axis. Histograms were prepared for each group and the distribution of myotube diameters was analyzed.

### 2.4. Analyses of mRNA Expression by Quantitative Polymerase Chain Reaction (qPCR)

mRNA expression of myogenic regulator factors, Gln transporters, and glutaminolysis-related enzymes was analyzed by qPCR to investigate changes in targeted gene expression. Total RNA was extracted from C2C12 myotubes using ISOGEN (NIPPON GENE, Tokyo, Japan), according to the manufacturer’s protocol. The concentration of total RNA was measured using NanoDrop Lite (Thermo Fisher Scientific Inc.). First-strand cDNA was synthesized from 1 μg of total RNA using a Superscript IV pre-amplification system (Thermo Fisher Scientific Inc.). cDNA was then amplified by real-time qPCR (RT-qPCR) with primer pairs designed using Primer3Plus (Whitehead Institute for Biomedical Research, Cambridge, MA, USA). PCR primer pairs were obtained from Eurofins Scientific (Luxembourg, UK). The sequences of the mouse PCR primer pairs were described in Table 1. RT-qPCR was performed using SsoAdvanced SYBR Green Supermix (Bio-Rad Laboratories Inc., Hercules, CA, USA) using a CFX Connect Real-Time PCR System (Bio-Rad Laboratories Inc.). The relative normalized gene expression, determined using the ΔΔCt method, was analyzed using Bio-Rad CFX Manager 3.1 (BioRad Laboratories Inc.). The relative amount of each transcript was normalized to the amount of Rn18s (18S ribosomal RNA) transcripts.

The mRNA expression of four types of Gln transporters—Asct2 (alanine, serine, cysteine, threonine transporter 2), Lat1 (L-type amino acid transporter 1), Snat1 (sodium-coupled neutral amino acid transporter 1), and Snat2—and six types of glutaminolysis-related enzymes—Gls1 (glutaminase 1), Gls2, Glul (Glu-ammonia ligase), Glud1 (Glu dehydrogenase 1), Gpt2 (Glu pyruvate transaminase 2), and Bcat2 (branched chain amino acid transaminase 2)—was analyzed in C2C12 myoblasts and on days 0, 4, and 7 post-differentiation.

### 2.5. Analysis of Protein Expression by Western Blotting

Western Blotting analyzed the change in the targeted protein’s expression. C2C12 myoblasts were cultured in the presence of 0, 1, 2, or 4 mM L-Gln or with or without CB-839 and A-485. C2C12 myotubes were lysed in lysis buffer (150 mM NaCl/50 mM Tris-HCl (pH7.4)/1 mM EDTA/1% (*v*/*v*) Triton-X) containing proteinase inhibitor (50 µg/mL leupeptin, 1.5 µM pepstatin, 1 mM PMSF; Abcam, Tokyo, Japan) and complete phosphatase inhibitor cocktail I (Abcam). Whole cell lysates were centrifuged at 12,000× *g* for 10 min, and the supernatant was collected. The protein concentration of the supernatant was measured using a bicinchoninic acid (BCA) protein assay kit (Thermo Fisher Scientific Inc.). Next, 6 µg of protein in each sample was subjected to sodium dodecyl sulfate-polyacrylamide gel electrophoresis (SDS-PAGE) with a 13% polyacrylamide gel and transferred onto polyvinylidene difluoride membranes (Merck Millipore Ltd., Cork, Ireland). The membranes were blocked with 5% dry milk in PBS with 0.05% Tween-20 (PBS-T) or 5% BSA in TBS with 0.05% Tween-20 (TBS-T) and incubated with primary antibodies at 4 °C overnight. Membranes were incubated with the corresponding secondary antibody in 1.5% skim milk in PBS-T or 1.5% BSA in TBS-T and developed with ECL Prime Western blotting Detection Reagent (Cytiva Inc., Tokyo, Japan) using ChemiDoc XRS+ (Bio-Rad Laboratories Inc.). Primary antibodies against Histone H3 (15–17 kDa; Protein Tech Inc., Chicago, IL, USA), Acetyl-Histone H3 (Lys27) (17 kDa; Cell Signaling Technology Inc., Danvers, MA, USA), and β-actin (43 kDa; Santa Cruz Biotechnology Inc., Dallas, TX, USA) were used.

### 2.6. Statistical Analysis

All statistical analyses were performed using the R software program (ver. 4.5.1, Posit Public Benefit Inc., Boston, MA, USA) and GraphPad Prism 10 software program (version 10.5.0 (774), GraphPad Software, San Diego, CA, USA). Normality of the data was assessed using the Shapiro–Wilk test, and equality of variance was evaluated using the Brown-Forsythe test for one-way ANOVA. For comparisons of three or more groups, one-way analysis of variance (ANOVA) was performed if normality and equal variance were satisfied. Post hoc multiple comparisons were conducted using Tukey’s test. Tukey’s test was used to compare all possible pairs of group means. Data were expressed as the mean ± standard error of the mean (SE), with individual data points shown. The significance of differences was analyzed, and *p* values of < 0.05 were considered statistically significant.

## 3. Results

### 3.1. Gln Enhances the Differentiation of C2C12 Myoblasts

**Figure 1:**
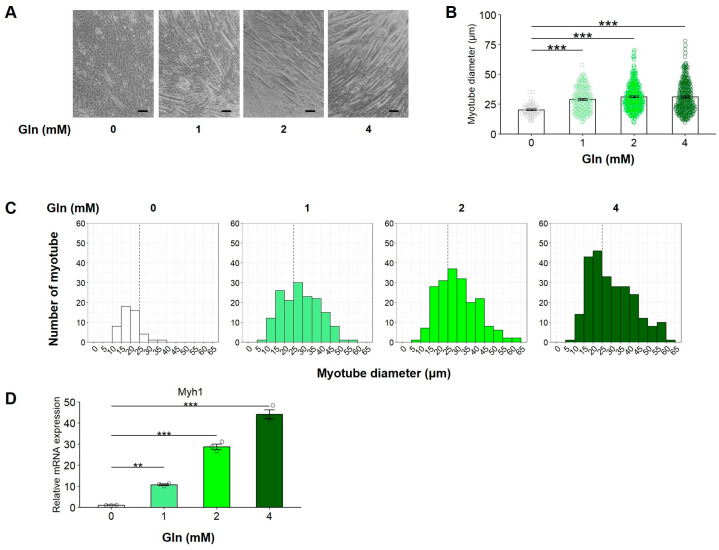
The effect of Gln on C2C12 myogenic differentiation. (**A**) C2C12 myoblasts were cultured in DM containing 0, 1, 2, or 4 mM L-Gln. Images show representative microscopic image. The scale bar indicates 100 µm. (**B**) Myotube diameter was measured using the ImageJ software program. The average of all measured myotube diameter was calculated. The data are expressed as the mean ± SE of counts from all the myotubes of nine microscopic images (0 mM: n = 48, 1 mM: n =160, 2 mM: n = 199, 4 mM: n = 252), collected from three independent wells, with individual data points. (**C**) The histogram shows the frequency distribution of myotube diameters using with 5-µm-wide bins. (**D**) The mRNA expression of Myh1 was analyzed by RT-qPCR. The data are expressed as the mean ± SE of three independent cultures, with individual data points. The Rn18s gene was used for normalization. Asterisks indicate significant differences between two groups: ** *p* < 0.01 and *** *p* < 0.001 vs. 0 mM L-Gln by a one-way analysis of variance (ANOVA) followed by Tukey–Kramer’s test.

To assess the effect of Gln on C2C12 myogenic differentiation, C2C12 myoblasts were cultured in a DM containing 0, 1, 2, or 4 mM L-Gln. Gln promoted myotube formation in a concentration-dependent manner and shifted the distribution of myotube diameters toward larger values (Figure 1A–C), accompanied by upregulation of Myh1 (Myosin heavy chain 1 (MyHC1) mRNA expression compared to the control group (Figure 1D)). These results suggest that Gln enhances C2C12 myoblast differentiation.

### 3.2. mRNA Expression of Gln Transporters and Glutaminolysis-Related Enzymes Increases During C2C12 Myogenic Differentiation

**Figure 2:**
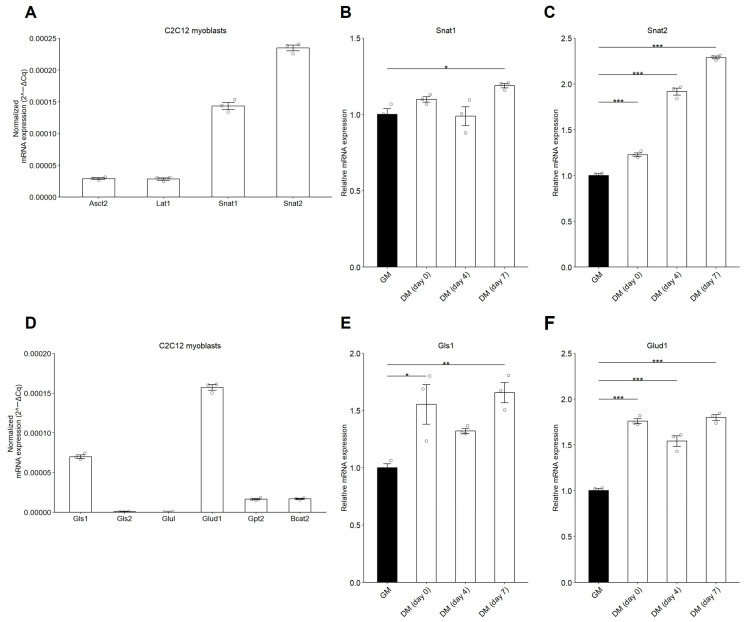
mRNA expression of Gln transporters and glutaminolysis-related enzymes in C2C12 myoblast differentiation. The mRNA expression of Gln transporters (**A**–**C**) or glutaminolysis-related enzymes (**D**–**F**) in C2C12 myoblasts was analyzed by RT-qPCR. C2C12 myoblasts were cultured in GM or DM. Total RNA was extracted, and the mRNA expression (2^−ΔCq^) of Gln transporters, including Asct2, Lat1, Snat1, and Snat2, in myoblasts was analyzed (**A**), and the relative mRNA expression (2^−ΔΔCq^) of Snat1 (**B**) and Snat2 (**C**) were analyzed during myoblast differentiation. The mRNA expression (2^−ΔCq^) of glutaminolysis-related enzymes, including Gls1, Gls2, Glul, Glud1, Gpt2, and Bcat2, in myoblasts was analyzed (**D**), and the relative mRNA expression (2^−ΔΔCq^) of Gls1 (**E**) and Glud1 (**F**) were analyzed during myoblast differentiation. The data are expressed as the mean ± SE of three independent cultures, with individual data points. The Rn18s gene was used for normalization. Asterisks indicate significant differences between two groups: * *p* < 0.05, ** *p* < 0.01 and *** *p* < 0.001 vs. GM by a one-way ANOVA followed by Tukey–Kramer’s test.

The mRNA expression of Snat2 was the highest among the Gln transporters and increased in a time-dependent manner during C2C12 myoblast differentiation (Figure 2A–C). When comparing the mRNA expression of Gls1 and Gls2, which catalyze the conversion of Gln into Glu, with that of Glul1, which is responsible for the reverse conversion of Glu into Gln, the mRNA expression of Gls1 was the highest (Figure 2D), and its expression was significantly elevated on days 0 and 7 post-differentiation compared to myoblasts (Figure 2E). Glud1, which catalyzes the conversion of Glu into α-KG, also showed higher expression than Gpt2 and Bcat2, which catalyze the conversion of Alanine or BCAA into Glu, respectively (Figure 2D). The mRNA expression of Glud1 increased on days 0 and 7 post-differentiation compared with that in myoblasts (Figure 2F). These results indicate that the mRNA expression levels of the Gln transporter Snat2 and glutaminolysis-related enzymes Gls1 and Glud1 are positively correlated with C2C12 myogenic differentiation.

### 3.3. Glutaminase Inhibition by CB-839 Suppresses Gln-Dependent Myotube Formation in C2C12 Myoblasts

**Figure 3:**
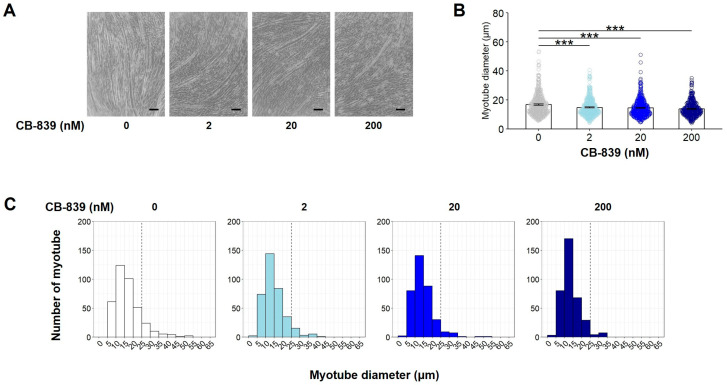
Effect of glutaminase inhibition on C2C12 myogenic differentiation. C2C12 myoblasts were cultured with the GLS inhibitor CB-839 (0, 2, 20, or 200 nM) in the presence of Gln. The representative microscopic images (**A**), average of all measured myotube diameter (**B**), and the histogram of the frequency distribution of myotube diameters using with 5 µm-wide bins (**C**). (**A**) Scale indicates 100 µm. (**B**) The data are expressed as the mean ± SE of counts from all the myotubes of nine microscopic images (0 nM: n = 383, 2 nM: n = 363, 20 nM: n = 360, 200 nM: n = 361), collected from three independent wells, with individual data points. Asterisks indicate significant differences between two groups: *** *p* < 0.001 vs. 0 nM CB-839 by a one-way ANOVA followed by Tukey–Kramer’s test. (**C**) The histogram shows the frequency distribution of myotube diameters using with 5-µm-wide bins.

To determine whether or not glutaminolysis plays a physiological role during C2C12 myoblast differentiation, C2C12 myoblasts were cultured with the GLS inhibitor CB-839 (0, 2, 20, or 200 nM), which selectively inhibits GLS and thereby blocks the initial reaction of glutaminolysis [24]. As shown in Figure 3A–C, the number of myotubes, particularly those with diameters greater than 15 μm, was reduced by CB-839 treatment compared to the control group. These results indicated that GLS-mediated glutaminolysis can accelerate C2C12 myoblast differentiation.

### 3.4. Glu Restores the Inhibition of C2C12 Myotube Formation Caused by Gln Deficiency and Gls Inactivation

**Figure 4:**
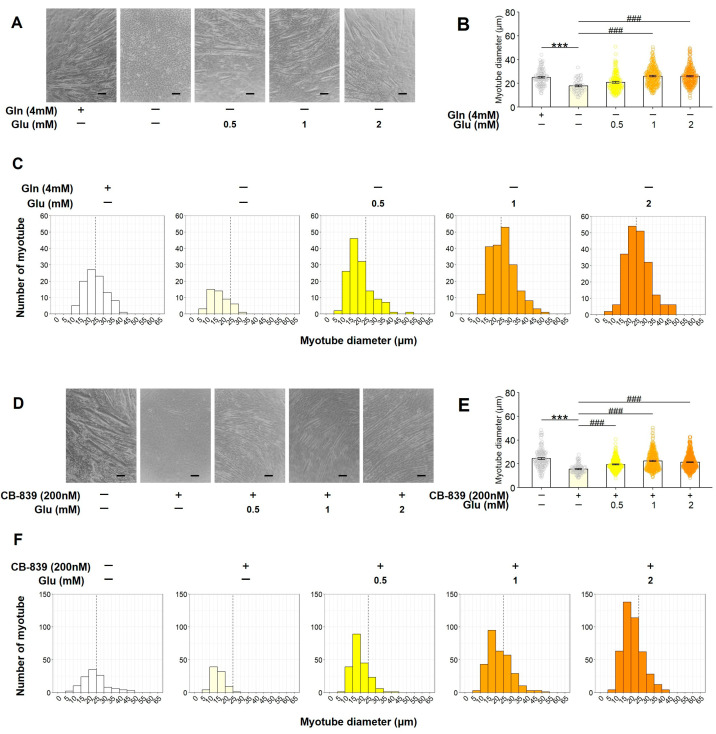
Compensatory effect of Glu on C2C12 myogenic differentiation suppressed by Gln deficiency or GLS inhibition. C2C12 myoblasts were cultured with Glu (0, 0.5, 1, and 2 mM) under Gln-deficient conditions (**A**–**C**) or in the presence of CB-839 (**D**–**F**). The representative microscopic images (**A**,**D**), average of all measured myotube diameter (**B**,**E**), and the histogram of the frequency distribution of myotube diameters using with 5-µm-wide bins (**C**,**F**). (**A**,**D**) Scale indicates 100 µm. (**B**,**E**) The data are expressed as the mean ± SE of counts from all the myotubes of nine microscopic images ((**B**); Control: n = 97, 0 mM Glu: n = 48, 0.5 mM Glu: n = 138, 1 mM Glu: n = 204, 2 mM Glu: n = 206) ((**E**); Control: n = 118, 0 mM Glu: n = 85, 0.5 mM Glu: n = 205, 1 mM Glu: n = 307, 2 mM Glu: n = 425), collected from three independent wells, with individual data points. Significant differences between two groups were indicated as follows: *** *p* < 0.001 vs. Cont. and ### *p* < 0.001 vs. Gln deficiency or CB-839 alone by a one-way ANOVA followed by Tukey–Kramer’s test.

We investigated whether or not Glu, a metabolite generated from Gln during glutaminolysis, could restore myotube formation suppressed by Gln deficiency or GLS inhibition. C2C12 myoblasts were cultured with Glu (0, 0.5, 1, and 2 mM) under Gln-deficient conditions or after treatment with CB-839 (200 nM). Gln deficiency markedly suppressed myotube formation, whereas Glu supplementation significantly restored myotube formation (Figure 4A–C). Similarly, CB-839 treatment in the presence of Gln suppressed myotube formation, which was reversed by Glu supplementation (Figure 4D–F). These results demonstrate that Glu, an intermediate of glutaminolysis, can restore C2C12 myotube formation, which is inhibited by blocking glutaminolysis.

### 3.5. DM-α-KG Restores the Inhibition of C2C12 Myotube Formation Caused by Gln Deficiency or GLS Inactivation

**Figure 5:**
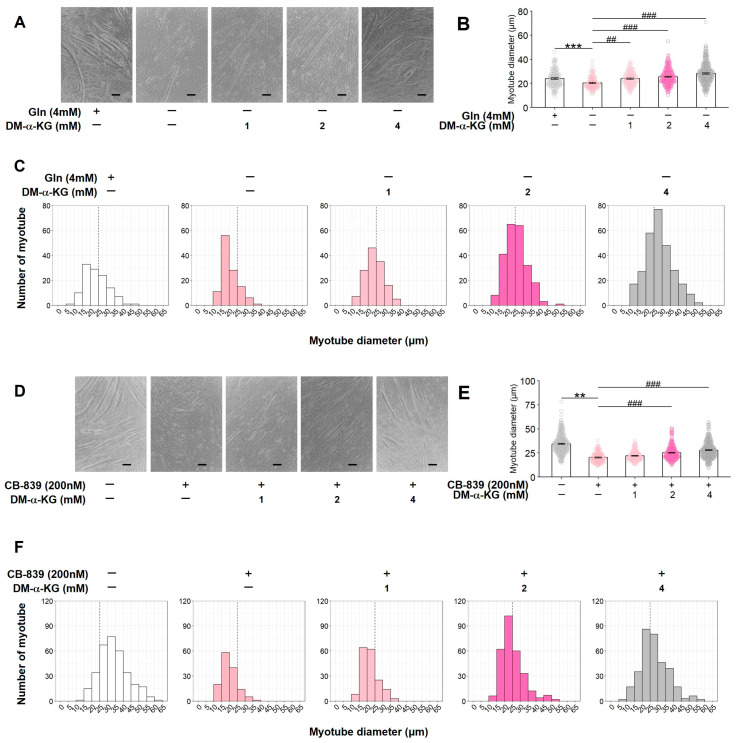
The compensatory effect of DM-α-KG on C2C12 myogenic differentiation suppressed by Gln deficiency or GLS inactivation. C2C12 myoblasts were cultured with DM-α-KG (0, 1, 2, or 4 mM) under Gln-deficient conditions (**A**–**C**) or in the presence of CB-839 (**D**–**F**). The representative microscopic images (**A**,**D**), average of all measured myotube diameter (**B**,**E**), and the histogram of the frequency distribution of myotube diameters using with 5-µm-wide bins (**C**,**F**). (**A**,**D**) Scale indicates 100 µm. (**B**,**E**) The data are expressed as the mean ± SE of counts from all the myotubes of nine microscopic images ((**B**); Control: n = 120, 0 mM DM-α-KG: n = 117, 1 mM DM-α-KG: n = 137, 2 mM DM-α-KG: n = 232, 4 mM DM-α-KG: n = 284) ((**E**); Control: n = 333, 0 mM DM-α-KG: n = 138, 1 mM DM-α-KG: n = 176, 2 mM DM-α-KG: n = 288, 4 mM DM-α-KG: n = 333), collected from three independent wells, with individual data points. Significant differences between two groups were indicated as follows: ** *p* < 0.01 and *** *p* < 0.001 vs. Cont., and ## *p* < 0.01 and ### *p* < 0.001 vs. Gln deficiency or CB-839 alone by a one-way ANOVA followed by Tukey–Kramer’s test.

During glutaminolysis, Glu converted from Gln is metabolized to α-KG by GLUD1. To experimentally assess the effect of α-KG, DM-α-KG (0, 1, 2, or 4 mM), a cell-permeable analog of α-KG, was added to C2C12 myoblast cultures under Gln-deficient conditions or after treatment with CB-839 (200 nM). Gln deficiency and CB-839 treatment in the presence of Gln significantly suppressed myotube formation, whereas DM-α-KG supplementation restored myotube formation inhibited by either Gln deficiency (Figure 5A–C) or GLS inhibition (Figure 5D–F) in a concentration-dependent manner. These results indicate that α-KG, an intermediate of glutaminolysis, can restore C2C12 myotube formation, which is suppressed by blocking glutaminolysis.

### 3.6. Glutaminolysis Modulates the mRNA Expression of Myog During C2C12 Myogenic Differentiation

**Figure 6:**
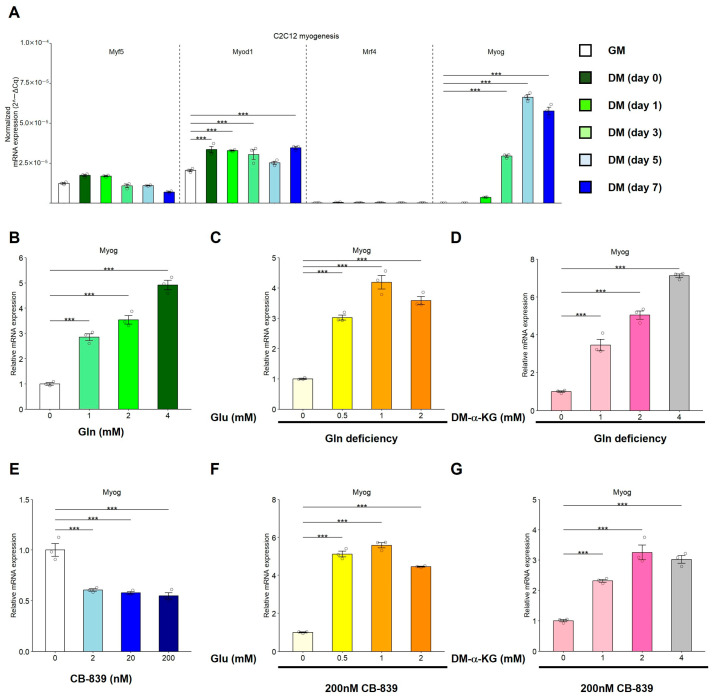
Glutaminolysis regulates the mRNA expression of Myog. (**A**) C2C12 myoblasts were cultured in GM or DM. The mRNA expression (2^−ΔCq^) of muscle regulatory factors, Myf5, Myod1, Mrf4, and Myog, was analyzed by RT-qPCR. (**B**–**G**) The mRNA expression (2^−ΔΔCq^) of Myog was analyzed in C2C12 myotubes cultured in the presence of Gln (0, 1, 2, or 4 mM; (**B**), in the presence of Glu (0, 0.5, 1, and 2 mM) under Gln deficiency (**C**), in the presence of DM-α-KG (0, 1, 2, or 4 mM) under Gln deficiency (**D**), in the presence of CB-839 (200 nM; (**E**)), in the presence of Glu (0, 0.5, 1, and 2 mM) and CB-839 (200 nM; (**F**)), and in the presence of DM-α-KG (0, 1, 2, or 4 mM) and CB-839 (200 nM; (**G**)). The data are expressed as the mean ± SE of three independent cultures, with individual data points. The Rn18s gene was used for normalization. Asterisks indicate significant differences between two groups: *** *p* < 0.001 vs. GM by a one-way ANOVA followed by Tukey–Kramer’s test.

To examine the mechanism underlying myogenic regulation by glutaminolysis, we analyzed the mRNA expression of MRFs, including Myf5, Myod1, Mrf4, and Myog. Myog mRNA expression showed the greatest increase during C2C12 myogenic differentiation in the presence of Gln, mRNA expression of Myog showed the greatest increase (Figure 6A). Next, C2C12 myoblasts were cultured in DM containing 0, 1, 2, or 4 mM L-Gln or 0, 2, 20, or 200 nM CB-839 for 5 days, and mRNA expression was analyzed. Gln significantly upregulated Myog mRNA expression of Myog in a concentration-dependent manner (Figure 6B). Under Gln-deficient conditions, supplementation with Glu or DM-α-KG significantly restored the mRNA expression of Myog (Figure 6C,D). In contrast, CB-839 treatment in the presence of Gln significantly downregulated the mRNA expression of Myog, whereas Myog upregulation induced by Glu or DM-α-KG supplementation was not affected by CB-839 treatment (Figure 6E–G). These results indicated that glutaminolysis regulates Myog expression during C2C12 myogenic differentiation.

### 3.7. Catalytic Inhibition of CBP/p300 Suppresses C2C12 Myogenic Differentiation in the Presence of Gln Through Reduction of H3K27 Acetylation

**Figure 7:**
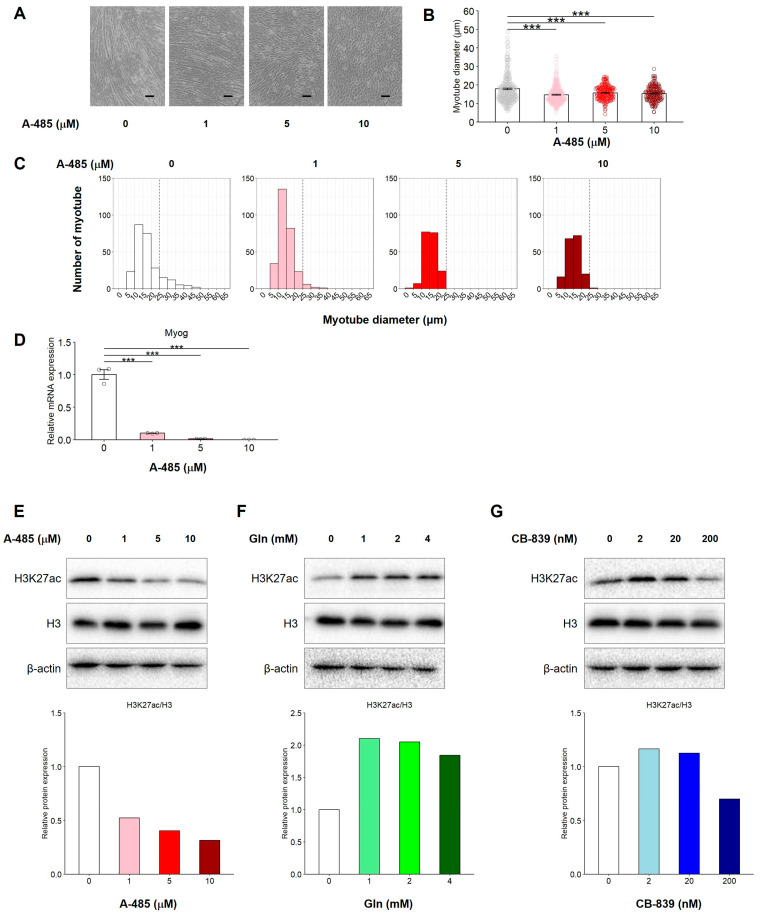
Catalytic inhibition of CBP/p300 suppresses C2C12 myogenic differentiation by reducing acetylation of Histone H3K27. C2C12 myoblasts were cultured with A-485 (0, 1, 5, or 10 µM), a CBP/p300 catalytic inhibitor, in the presence of Gln. The representative microscopic images (**A**), average of all measured myotube diameter (**B**), the histogram of the frequency distribution of myotube diameters using with 5-µm-wide bins (**C**), and the mRNA expression (2^−ΔΔCq^) of Myog (**D**). (**A**) Scale indicates 100 µm. (**B**) The data are expressed as the mean ± SE of counts from all the myotubes of nine microscopic images (0 µM: n = 251, 1 µM: n = 283, 5 µM: n = 185, 10 µM: n = 177), collected from three independent wells, with individual data points. (**D**) The data are expressed as the mean ± SE of three independent cultures, with individual data points. The Rn18s gene was used for normalization. (**E**–**G**) C2C12 myoblasts were cultured in DM containing 0, 1, 2, or 4 mM L-Gln with A-485 (0, 1, 5, or 10 µM) or CB-839 (0, 2, 20, or 200 nM) for 24 h. Whole lysates were collected, and the protein expression of H3K27ac, H3 and β-actin was detected by Western blotting. Upper images of the blots are shown in indicated. Lower images indicated the relative intensity of H3K27ac protein expression. Uncropped raw blot images are shown in Supplementary Figure S1. Asterisks indicate significant differences between two groups: *** *p* < 0.001 vs. 0 µM A-485 by a one-way ANOVA followed by Tukey–Kramer’s test.

A potential explanation for the changes in gene expression regulated by glutaminolysis may involve epigenetic modifications, as intermediates of the TCA cycle serve as substrates for histone- and DNA-modifying enzymes [25]. To test whether or not the histone acetyltransferase activity of CBP/p300 contributes to Myog transcriptional regulation, C2C12 myoblasts were cultured with A-485, a CBP/p300 catalytic inhibitor of Gln. As shown in Figure 7A–C, A-485 treatment decreased the number of myotubes in a concentration-dependent manner, and the proportion of myotubes with diameters less than 15 μm increased. In addition, the mRNA expression of Myog was significantly downregulated by A-485 compared with that in the control group (Figure 7D). An analysis of H3K27 acetylation (H3K27ac) levels by Western blotting showed that Gln increased H3K27ac levels in a concentration-dependent manner (Figure 7E). However, treatment with CB-839 or A-485 in the presence of Gln reduced H3K27ac levels (Figure 7F,G), which correlated with the inhibition of myotube formation (Figure 3A and Figure 7A). These data suggest that the inhibition of CBP/p300 suppresses C2C12 myogenic differentiation in the presence of Gln by reducing H3K27ac levels.

## 4. Discussion

In this study, we investigated whether Gln regulates Myog expression, an essential gene for myogenic differentiation, through p300-dependent H3 acetylation. Treatment with 4 mM Gln increased myotube formation, increasing diameter by 50%, Myog gene expression by 5-fold, and H3K27 acetylation level by 2-fold. Furthermore, Gln metabolites upregulated Myog gene expression under Gln deficiency or CB-839 treatment by several fold. Dohl et al. reported that Gln deficiency inhibited the proliferation and differentiation of C2C12 myoblasts and induced cell death [26]. The in vivo circulation dynamics and organ interactions of Gln have not been considered in this study; therefore, further investigation is required regarding Gln’s muscle-forming regulatory effects within the body.

The present study demonstrated that Gln promotes C2C12 myotube formation, whereas Gln deprivation suppresses it (Figure 1 and Figure 3). Among Gln transporters, Snat1 and Snat2 were highly expressed compared to Asct2 and Lat1 in C2C12 myoblasts, and Snat2 expression was significantly upregulated during myogenic differentiation (Figure 2A–C). These findings suggest that extracellular Gln is primarily imported through SNAT transporters, thereby exerting myogenic effects. Thus, Gln enhances the myogenic potential of stem and progenitor cells, thereby contributing to muscle regeneration.

**Figure 8:**
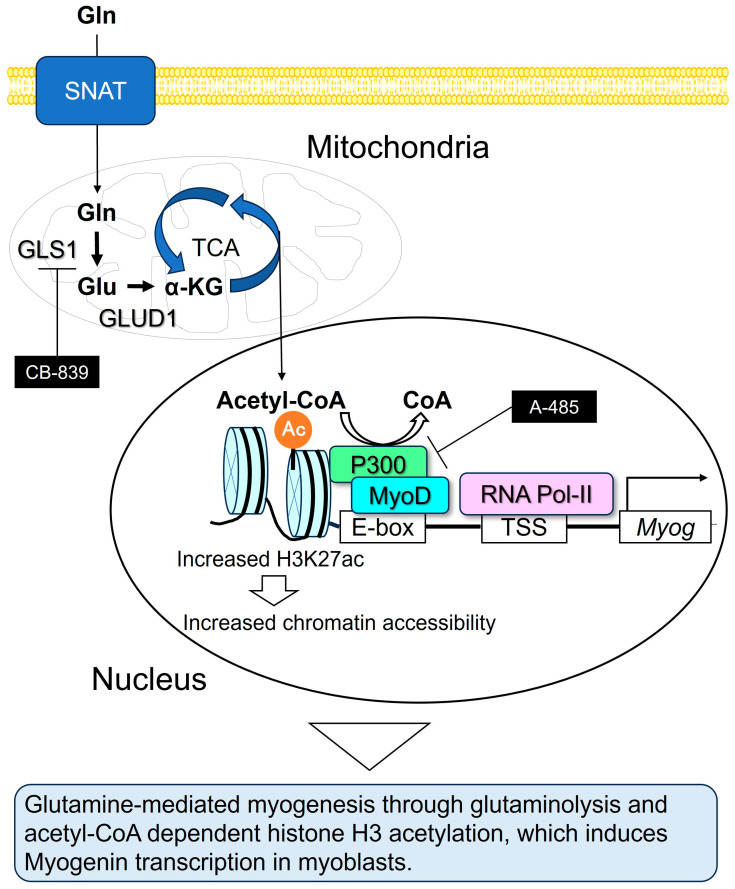
Schematic model illustrating the effects of Gln on myoblast differentiation. Gln is incorporated into cells through SNAT transporters and sequentially converted to Glu by GLS1 and α-KG by GLUD1. Then, α-KG metabolizes acetyl-CoA via TCA cycle, and acetyl-CoA mediates histone H3 acetylation by activating HAT activity of CBP/p300 in the Myog promoter, resulting in upregulating Myog expression and myogenic differentiation.

Skeletal muscle proteins are composed of multiple amino acids, classified as either essential (e.g., lysine, leucine) or non-essential (e.g., Gln, glycine, and serine). Gln is the most abundant amino acid in the human body, with the majority stored in skeletal muscle [1]. Lysine has been reported to promote muscle stem cell proliferation and prevent apoptosis by activating the mTORC1 signaling pathway [27]. Primary myoblasts also require extracellular supplementation of non-essential amino acids such as serine and glycine for proliferation [28]. Furthermore, leucine-enriched diets during recovery from cryo-injury suppress the forkhead box O3a (FoxO3a) pathway—a key regulator of muscle protein degradation—thereby accelerating recovery of myofiber size and contractile strength in both young and aged rats [29,30]. Collectively, these observations suggest that both essential and nonessential amino acids are required for efficient muscle regeneration. Our findings provide new insights by identifying Gln, a non-essential amino acid, as a critical factor for myogenic differentiation accompanied by increased Myog expression and elevated H3K27 acetylation (Figure 8). Since mTOR is an essential factor for protein synthesis, its activity is considered indispensable for protein expression following transcriptional activation. In future experiments, we plan to investigate the involvement of mTOR in glutamine-induced myogenin transcriptional activation, protein synthesis, and myogenic differentiation.

During myoblast differentiation, Myog functions as a key transcription factor that regulates the expression of muscle-specific genes, such as myosin heavy chain, troponin, and muscle creatine kinase, by binding to E-box DNA motifs (CANNTG) near transcription start sites [31,32,33]. Myog expression is defective in MyoD1-deficient primary myoblasts [34], indicating that MyoD acts as an upstream regulator. MyoD serves as a master transcription factor that directs progenitor cells toward the myogenic lineage and induces Myog transcription for further differentiation. MyoD forms complexes with transcriptional coactivators, such as CBP/p300, which possess histone acetyltransferase (HAT) activity that acetylates H3K27. This acetylation is essential for Myog transcription and subsequent stages of myogenic differentiation [35,36,37]. Based on these findings, we investigated the relationship between MyoD and its downstream target Myog to elucidate the molecular mechanism by which Gln promotes myogenesis. In the present study, we discovered that H3K27 acetylation levels were increased by Gln treatment, which was accompanied by the upregulation of Myog expression during myogenic differentiation. Pharmacological inhibition of CBP/p300 reduced both H3K27ac and Myog mRNA levels, resulting in impaired myotube formation (Figure 7A–E).

In previous reports, glutaminolysis-dependent histone H3 acetylation was shown to induce chondrogenic gene (aggrecan and type 2 collagen) transcription in growth plate chondrocytes [11]. Glucose and glutamine have been reported as primary sources of acetyl-CoA [6]. Energy production in skeletal muscle stem cells relies on glycolysis during the proliferation phase but on oxidative phosphorylation during differentiation [38,39]. Previous in vitro research demonstrated that protein acetylation during muscle differentiation depends on glutamine rather than glucose [40]. These prior studies support the notion that the increase in acetylation is attributable to acetyl-CoA derived from glutaminolysis. However, acetyl-CoA is a central metabolite with multiple sources, including fatty acids; therefore, we need to investigate the different source-dependent histone H3 acetylation in C2C12 myoblasts. Confirmation of levels of acetyl-CoA and CBP/p300-mediated transcriptional activation of Myog by performing an H3K27ac ChIP-qPCR analysis at the Myog promoter in C2C12 myoblasts is required to elucidate the precise mechanisms underlying glutaminolysis-dependent histone H3 acetylation of specific myogenic targets.

Several studies have reported linking glutaminolysis and histone modifications, indicating that Gln-derived acetyl-CoA contributes to histone acetylation and epigenetic regulation of gene expression [10,11,12]. Consistent with these studies, our results show that H3K27 acetylation by CBP/p300 mediates glutaminolysis-related myogenic differentiation in C2C12 cells. Blocking glutaminolysis, either by Gln deprivation or treatment with the GLS inhibitor CB-839, decreased H3K27ac levels during differentiation, leading to suppression of myotube formation and downregulation of Myog expression (Figure 7F,G). Remarkably, supplementation with Glu or DM-α-KG restored myotube formation, even in the absence of Gln or in the presence of CB-839. These findings indicate that Gln, Glu, and α-KG are sequentially metabolized to acetyl-CoA during glutaminolysis, which serves as a key substrate for histone acetylation and MyoD-dependent Myog transcription. Further studies are warranted to clarify how intracellular acetyl-CoA levels regulate the transcriptional and epigenetic activation of Myog during myogenic differentiation.

In another part of this study, Gls1, encoding the enzyme that converts Gln to Glu, was expressed at higher levels than Gls2 or Glul (which catalyzes the reverse reaction) in C2C12 myoblasts (Figure 2D). Similarly, Glud1, which encodes Glu dehydrogenase that converts Glu to α-KG [41], showed higher expression than Gpt2 or Bcat2, which catalyze the reverse reaction (Figure 2D). Notably, Gls1 and Glud1 expressions progressively increased during myogenesis (Figure 2E,F). These findings suggest that the activation of glutaminolysis occurs during myogenic differentiation, supported by the elevated expression of Gls1 and Glud1. In contrast, plasma Gln levels and glutaminolytic activity decrease under conditions of physical inactivity [42,43]; therefore, Gln deficiency may contribute to skeletal muscle atrophy by impairing Myog transcriptional activation. Further studies are needed to determine whether or not Gln supplementation can reverse atrophic phenotypes in inactive models, such as hindlimb unloading.

## 5. Conclusions

This study demonstrates that Gln facilitates myogenic differentiation through epigenetic regulation of MyoD-dependent Myog transcription in myoblasts. This epigenetic mechanism is likely mediated by acetyl-CoA generated from glutaminolysis. Skeletal muscle injury induces regeneration, in part by enhancing plasma Gln-driven myogenesis. In cases of strenuous exercise or resistance training, Gln supplementation is beneficial for promoting muscle regeneration and myogenesis through the epigenetic activation of myogenic transcription factors. Further studies will examine the effects of Gln supplementation during resistance training in athletes, suggesting that it may be an effective strategy to accelerate muscle regeneration.

## Figures and Tables

**Table 1:** Primer sequences for qPCR.

Gene	Forward	Reverse
*Rn18s*	5′-tcaagaacgaaagtcggagg-3′	5′-ggacatctaagggcatcac-3′
*Myh1*	5′-gagggacagttcatcgatagcaa-3′	5′-gggccaacttgtcatctctcat-3′
*Asct2*	5′-tccagcgggagatcaattcaa-3′	5′-gacgatagcgaagaccacca-3′
*Lat1*	5′-ctggatcgagctgctcatc-3′	5′-gttcacagctgtgaggagc-3′
*Snat1*	5′-tccatgactctcgaccagaac-3′	5′-cgaaggcgatggttggtaaagc-3′
*Snat2*	5′-ttgctcgctgctctctttgg-3′	5′-cacgatctcggagtaggtatgc-3′
*Gls1*	5′-caacgtcagatggtgtcatgc-3′	5′-cctccagactgctttttagcac-3′
*Gls2*	5′-acaagatggctgggaacgaa-3′	5′-tgaggtaatagccgat-3′
*Glul*	5′-tggctggtcaacttga-3′	5′-tcaaaaggcccgcttt-3′
*Glud1*	5′-ttggtcctggcattgatgtg-3′	5′-taacacaggcatgcgcattg-3′
*Gpt2*	5′-aagaaggagcgcatgcaatc-3′	5′-atttgcttggtggctgctac-3′
*Bcat2*	5′-cggacccttcattcgtcaga-3′	5′-ccatagttcccccccaactt-3′
*Myf5*	5′-tgaatgtaacagccctgtctggtc-3′	5′-cgtgatagataagtccggagctgg-3′
*Myod1*	5′-agcatcacagtggcgactca-3′	5′-ggccgctgtaatccatcat -3′
*Mrf4*	5′-ggccaagtgtttcggatcattc-3′	5′- ttccaaatgctggctgagttacttc-3′
*Myog*	5′-cccatggtgcccagtgaa-3′	5′-gcagatgtggggcgtctgta-3′

## Data Availability

The original contributions presented in the study are included in the article, and further inquiries can be directed to the corresponding author.

## Supplementary Materials

The following supporting information can be downloaded at: https://www.mdpi.com/article/10.3390/nu17233673/s1, Figure S1: Uncropped raw Western blot images of Figure 7E–G.
