# Supplementary material for: Glutamine Promotes Myogenesis in Myoblasts Through Glutaminolysis-Mediated Histone H3 Acetylation That Enhances Myogenin Transcription
Source: Nutrients. 2025 Nov 24;17(23):3673. doi: 10.3390/nu17233673 (PMC12693497; doi:10.3390/nu17233673)

Supplementary Figure S1.

In our laboratory, to conserve antibodies, we cut out the portion of the Western blot membrane corresponding to the molecular weight of the target protein and perform the antigen–antibody reaction only on that section.

Below, we present the original, uncropped, and unadjusted images. By comparing the two pictures, it can be understood that the figures in the paper were prepared by appropriately trimming these original blots.

A **Relative band images of western blotting in Figure 7E**

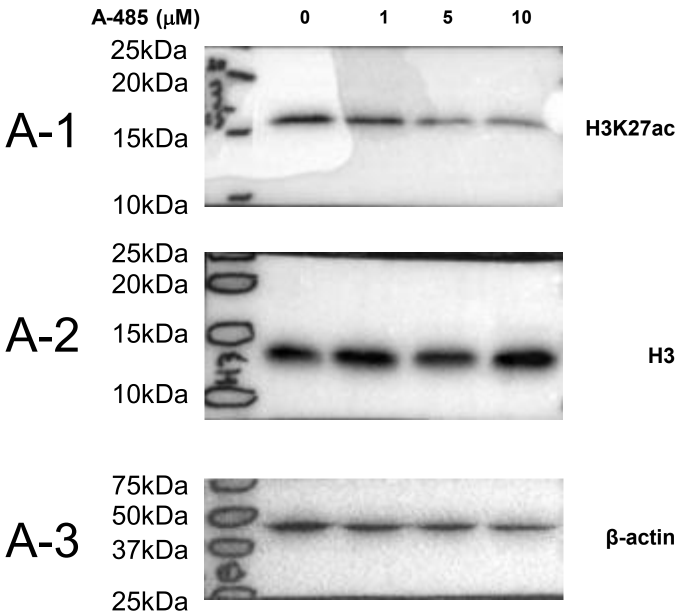

B **Relative band images of western blotting in Figure 7F**

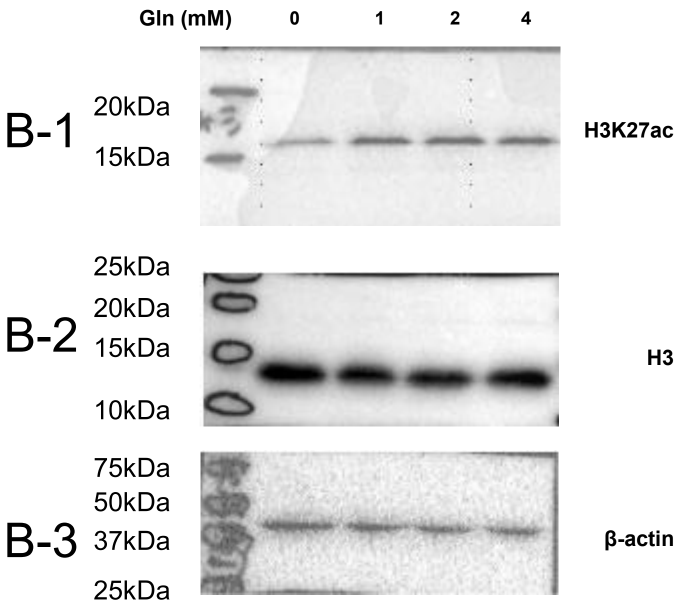

C **Relative band images of western blotting in Figure 7G**

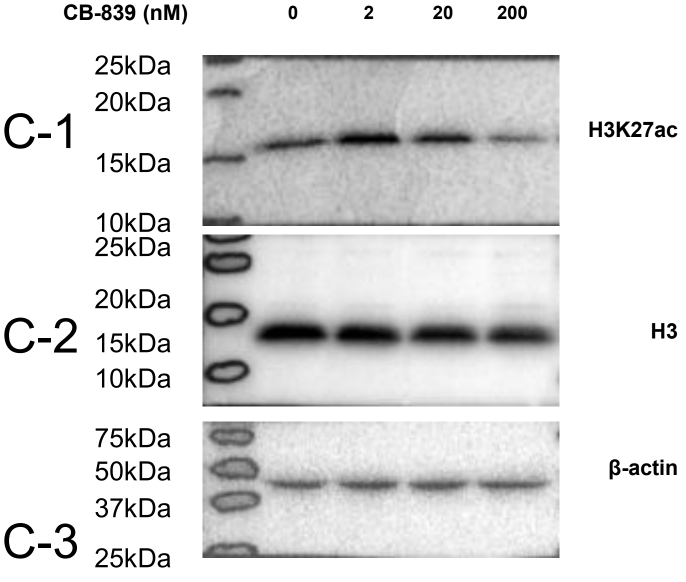

Supplement: Supplementary file 1 [file nutrients-17-03673-s001.zip › nutrients-3964729-supplementary.pdf]
